# Interspecific Hybridization of Transgenic *Brassica napus* and *Brassica rapa*—An Overview

**DOI:** 10.3390/genes13081442

**Published:** 2022-08-13

**Authors:** Soo-In Sohn, Senthil Kumar Thamilarasan, Subramani Pandian, Young-Ju Oh, Tae-Hun Ryu, Gang-Seob Lee, Eun-Kyoung Shin

**Affiliations:** 1 Department of Agricultural Biotechnology, National Institute of Agricultural Sciences, Rural Development Administration, Jeonju 54874, Korea; 2 Institute for Future Environment Ecology Co., Ltd., Jeonju 54883, Korea

**Keywords:** interspecific hybridization, *Brassica rapa*, *Brassica napus*, genetically modified crops, crossability, ploidy, backcross progenies

## Abstract

In nature, interspecific hybridization occurs frequently and can contribute to the production of new species or the introgression of beneficial adaptive features between species. It has great potential in agricultural systems to boost the process of targeted crop improvement. In the advent of genetically modified (GM) crops, it has a disadvantage that it involves the transgene escaping to unintended plants, which could result in non-specific weedy crops. Several crop species in the *Brassica* genus have close kinship: canola (*Brassica napus*) is an ancestral hybrid of *B. rapa* and *B. oleracea* and mustard species such as *B. juncea*, *B. carinata*, and *B. nigra* share common genomes. Hence, intraspecific hybridization among the *Brassica* species is most common, especially between *B. napus* and *B. rapa*. In general, interspecific hybrids cause numerous genetic and phenotypic changes in the parental lines. Consequently, their fitness and reproductive ability are also highly varied. In this review, we discuss the interspecific hybridization and reciprocal hybridization studies of *B. napus* and *B. rapa* and their potential in the controlled environment. Further, we address the fate of transgenes (herbicide resistance) and their ability to transfer to their progenies or generations. This could help us to understand the environmental influence of interspecific hybrids and how to effectively manage their transgene escape in the future.

## 1. Introduction

Globally, the cropping area of genetically modified (GM) crops has constantly increased since 1996 [1]. GM crops cause huge nuisances to the environment, such as super weeds and introgressive hybridization. Concerns regarding the environmental consequences of the release of transgenic crops have led to considerable research to reduce the degree of ambiguity surrounding the risk of transgene escape via hybridization [2]. While reports of hybridization in natural environments are the most conclusive proof that transgenes can escape by hybridization, they are insufficient to evaluate the complete frequency of hybridization [3]. On the other hand, reports of hybridization between crops and their relatives through artificial hand-pollination are valuable sources of information because they allow for the assessment of reproductive compatibility between species and the detection of undesirable species combinations. This helps us to conduct a conservative analysis of species that should be considered for their potential as targets for transgene escape in the local environment [2,4].

In this review, we aimed to discuss the potential transgene escape via interspecific hybrids in the genus *Brassica*, one of the important genera in the *Brassicaceae* family, which comprises 39 species [5]. It is mainly cultivated for its edible roots, stems, leaves, buds, flowers, mustard, and oilseeds [6]. Oilseed rape (*Brassica napus* L.) is an allotetraploid species that arose through a spontaneous hybridization of *Brassica rapa* L. and *Brassica oleracea* L. It has the complete diploid chromosome sets of the highly homologous A and C genomes of *B. rapa* and *B. oleracea,* respectively. Among the various GM crops, *B. napus* L. is widely cultivated and has a high potential for hybridization with the closely related *Brassica* species through interspecific hybridization. It can spontaneously hybridize with *B. rapa* in both greenhouse and field experiments [7,8,9,10,11,12,13,14]. One of the main issues in the cultivation of transgenic *B. napus* is that the transgene may have been transferred through hand pollination and/or spontaneously to their wild relatives/cultivars, with undesired ecological consequences that can increase the fitness and invasiveness of weedy populations [1]. Aside from that, GM crops and their transgenes spread via seed spillage during transportation and pollen-mediated gene transfers, resulting in feral populations [1,15]. If this occurs, weeds with GM traits may provide new and substantial weed control challenges [16]. The risk of crop genes transferring to weedy relatives is determined by their genetic and structural similarities as well as the strength of the transgenic selection in the weedy relative. The transfer of transgenes through introgression also depends on the fitness of the first and successive generations of hybrids [16,17,18]. However, the level of hybridization and introgression among the *Brassica* species is highly varied. Therefore, in this review, we provide an overview of a different combination of interspecific hybridization between transgenic *B. napus* and close relative *Brassica* species in controlled greenhouse conditions. In addition, we have discussed GM traits fitness in interspecific hybridization, further highlighted the fate of transgenes, and addressed the risk factors for cross-combination effects.

## 2. Interspecific Hybridization of Transgenic *B. napus* and *B. rapa*

Interspecific hybridization is a common and important evolutionary mechanism in the *Brassicaceae* family. Genome polyploidization, genome duplication, and gene flow maintenance may occur several times during evolution [19,20,21]. Parental cross or reciprocal interspecific hybridization and backcrossing are important factors that can result in significant differences in male and female fitness. It strongly suggests that the likelihood of gene transfer is influenced by a number of factors, including the origin of wild plants; genome constitution; population structure; mating system of the hybridizing plants; field experiment designs; weed control measures; several growing seasons; co-existing species; and the possibility of introgression [7,22,23,24].

### 2.1. Hybrid Generation: Brassica rapa (♀) × GM Brassica napus (♂)

**Figure 1:**
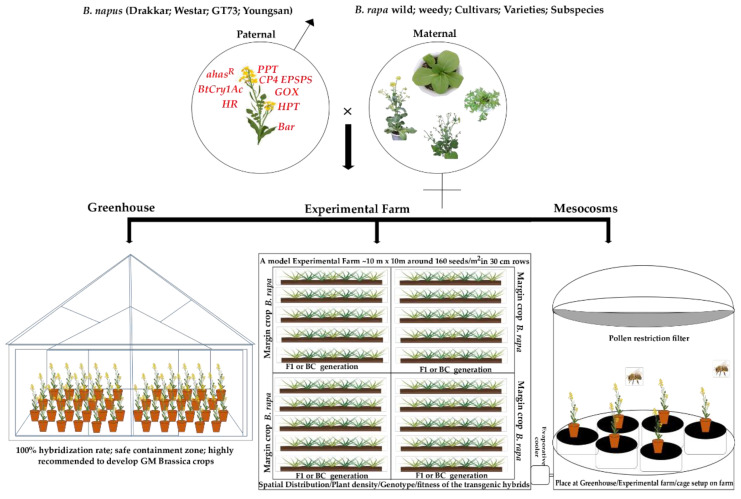
An overview of interspecific hybridization between *B. rapa* × GM *B. napus* through various conditions.

The crossability of weedy relatives or cultivated species of *B. rapa* (♀) and GM *B. napus* (♂) has been dependent on various environmental factors such as spatial distribution, maternal and paternal traits, and field or controlled conditions. Accordingly, various studies have reported that hybridization in controlled greenhouse circumstances will result in higher opportunities for genetically modified organism (GMO) crops and prevent controlled transgene flow in nature. An overview on interspecific hybridization between transgenic *B. napus* and *B. rapa* is represented in Figure 1 and Table 1. Initially, Vacher et al. [25] studied transgenic F_1_ hybrids with high fertility and backcrossing abilities under various ecological factors. It may enhance the fitness of transgenic hybrids and wild relatives. The high frequency (27%) occurred in areas with a higher plant density than usual (25%). However, the first-generation backcross and F_2_ hybrids might slow down the process of transgene spread with lower fitness. Likewise, F_1_ hybrids had higher fitness and silique per plant than backcross generations [26]. Pallett [27] has found a 5 to 100% hybridization rate between wild UK *B. rapa* and transgenic *B. napus* in optimum conditions. Consequently, the weedy population is highly variable when the hybridization is carried out, even in controlled conditions. Concerned with heterospecific pollination (removal of any co-flowering or likelihood that plants may interact via pollinators), they even observed matromorphs and apomicts (an asexual mode of reproduction; the ovule develops into seeds without involving meiosis and fertilization) due to parental combinations. In particular, the F_1_ generation had a higher percentage of C genome and transgene presence in all progenies than the backcross generation. Similarly, Vacher [28] found a predominantly out-crossing rate and added counter selection of the wild weedy phenotype population of UK *B. rapa* (♀) against transgenic *B. napus*.

The hybridization occurred due to flowering and for longer periods of time, which were likely to receive pollen from the transgenic trait. Naturally, the weedy plants had wider stems/stem diameters. Transgenes are involved in promoting flowering in nature. However, it produced a lower number of seeds. Also found, maternal weeds are less fit due to the longer period of flowering, which has a higher probability of hybridization with GM crops. Xiao et al. [31] extended different varieties of *B. rapa* to exhibit different levels of crossability index under controlled greenhouse conditions and compared them to spontaneous hybridization outcrossing. Due to pollen adhesion on the stigma and pollen tubes in the style, their numbers were reduced during self-pollination and were highly related to the genotypes of the parents. Likewise, broken or precocious germination was found in all the interspecific hybrids. Similarly, Sohn et al. [4] observed moderate crossability between *B. rapa* ssp. and transgenic GM *B. napus* through hand pollination under controlled conditions, with 100% crossability indices in F_1_ hybrids. Due to callus tissue formation during seed development and hormonal imbalance, the combination of the parental lines may cause precocious or cracked seeds, resulting in smaller sizes and affecting seed germination.

### 2.2. Backcross Generation: B. rapa (♀) × F_1_ (♂) (B. rapa × GM B. napus)

The transgenic F_1_ hybrids are likely to transfer the transgenes into backcrossing populations as seed parents of *B. rapa*. Initially, in field experiments, introgression of transgenic *B. napus* and weedy *B. rapa* were grown together. The weedy plants of *B. rapa* sp. have produced herbicide-tolerant BC_1_ generations with the same morphology and chromosomes as *B. rapa*, but they are more fertile and produced as early as the BC_1_ generation [42]. The phosphinothricin (PPT)-tolerant backcross generation had a lower ratio of monogenic segregation than the PPT-susceptible mendelian segregation ratio, indicating that the PPT-tolerant plants are homozygous or hemizygous. During the initial hybridization event with *B. rapa* as female parent and PPT-tolerant hybrid plants from backcrossed used as male parent transgene, the transgene transmission was reduced in the subsequent generations [35]. Furthermore, these results corroborate the transgene flow process and lower fitness level in the first generation or offspring or BC_1_ as a seed parent for *B. rapa* [26,43]. From another perspective, Halfhill et al. [11] used green fluorescent protein GFP/Bt as a tracking tool to assess the transgene flow and easy-to-use monitoring qualities of GFP with an agronomically significant transgenic. Though herbicide tolerance can be detected more effectively in a large number of plants than in GFP, this requires a visual assay of each individual for accurate screening [38]. In addition, the evidence of 12 transgenic at similar rates are the largest sample ever examined, in contrast to [35], two independent herbicide tolerant canola produced BC_1_ plants at dramatically different rates. After repeated backcrosses, Halfhill et al. [9] extended the work with other lines of weedy *B. napus* and *B. rapa* varieties to document gene flow and show that the resultant transgenic plants have fewer chromosomes and take on the morphological characteristics of their weedy *B. rapa* parent. Even though the hybridization rate was similar for both the weedy and cultivar of *B. rapa* when they were co-occurring, transgenic *B. napus* depends on density and spatial distribution [44]. Subsequently, he proposed to mitigate the gene flow and several factors in order to underestimate the actual frequency of gene flow from crop to weedy plants. Hence, the additive transgene has been used to locate the two copies of the transgene expression in homozygous individuals of canola as well as in hemizygous individuals (F_1_, BC_1_F_1_ and BC_2_F_2_). The F_1_ generation contained 95 to 97% of the genetic nature of *B. napus*, while subsequent backcross generations lost 15 to 29% of the genetic content in the BC_2_F_2_ bulk population [32]. This was followed by transgene mitigating *B. napus* containing the dwarfing gene, which demonstrated that the transgene mitigating (TM) strategy was effective in limiting seed production and thereby mitigating transgene flow from *B. napus* to *B. rapa*. In subsequent generations, the deleterious allele would only be expressed in homozygous individuals, which would strongly reduce its ability to decrease fitness [33].

In another study, transgene mitigating with additional genetic load and interspecific competition with wheat or more weed-like conditions was imposed, but still effective in limiting transgene persistence in weedy relatives [30]. Then, hybridization frequencies of 1 to 17% were observed with *B. napus* varieties and *B. rapa* [9], extended with multiple independent transformed lines, and several experimental conditions and locations were used to observe hybridization frequencies. The backcross frequencies are very low (0.074%) compared to expected (2.5%) but have a high level of potential to produce transgenic seeds [38]. Hence, backcrossing with a single transgenic event under a wide range of field conditions with competitive and non-competitive species, they found a lower vegetative growth rate and reduced from the triploid F_1_ generation to the diploid BC_2_F_2_ generation for the transgenic progenies of *B. rapa*. Subsequently, Vacher et al. [25] demonstrated that 1.4 times more seed in hybrids and backcross generations enhanced relative fitness under high herbivore pressure or selection pressure with more complex environments, using high fertilities and high backcrossing abilities of F_1_ hybrids. Sutherland et al. [45] agreed with the results, stimulating herbivore to their transgenic hybrids may increase the fitness and numbers of their progenies. However, the absence of herbivore pressure and continuous backcross generations maintain the physiological characteristics and decrease in fitness of transgenic hybrids in contrast to Halfhill et al. [9]. Another study showed remarkably similar growth and nitrogen utilization efficiency when compared to backcross generations of *B. napus* and *B. rapa* and transgenic *B. napus* F_1_ hybrids. These parameters, meanwhile, were less favorable than those of the wild relative, *B. rapa*, indicating that transgenic hybrids are less adapted to their natural environmental conditions [46]. In similar case, multiple transgenic (GT) lines were used with wild *B. rapa* the frequency of hybrids in BC_1_ progenies was higher than Halfhill et al. [11], but the segregation ratio was significantly deviated from BC_2_ to BC_3_ [10]. There are various factors affecting hybridization success irrespective of the presence or absence of transgenes that are less fit than the parental weed populations [40]. The transgene persistence was measured over six years under agro-environmental conditions, as claimed in the first report. In *B. rapa,* introgression may have a local gene pool which shows reduced fertility in progenies though the parents had normal fertility. However, continuous advanced backcross hybrid generation may reduce (6.2% lower) the fitness of hybrids over time [41]. On the other hand, herbicide drift *CP4* *EPSPS* selectively neutralizes transgenes, which does not affect the relative fitness relationship between the parental and backcross generation. Due to synchronizing the flowering time as early as possible, it may desynchronize from *B. napus* flowering time to reduce the potential gene flow. F_1_ hybrids may affect gene flow rates by preferentially pollinating with transgenic pollen rather than *B. rapa* pollen. It may be that sub-lethal application may be sufficient to alter the fitness and gene flow dynamics of transgenes [37].

## 3. Ploidy Determination for Transgenic Hybrids and Backcross Generation

Ploidy levels maintain desirable hybrid combinations during sexual reproduction in interspecific hybridization. During the hybridization process of a transgene from *B. napus* (2n = 38, AACC) to *B. rapa* (2n = 20, AA), a hybrid with (2n = 29, AAC) herbicide-resistant or transgenic lines was produced. Many researchers have observed that F_1_ hybrids produced from the hybridization of *B. rapa* *and B. napus* were triploid (AAC; 2n = 29) [4,9,11,35,45,47]. However, rather than a gene, the chromosome number determines an individual’s fitness in the backcross progenies [48]. Moreover, it was hypothesized that the loss of a C-chromosome during meiosis in backcross generation accounted for the lower transmission rate of a C chromosome in the BC_1_ generation [35,49,50]. Previously, Metz et al. [35] proposed that *B. rapa* transgenic individuals with 2n = 21 to 2n = 23 with transgenic TP2 produce AACC with a lower frequency of BC_2_, BC_3_, and BC_4_ populations by using only 1–4 herbicide-resistant individual plants, and that the frequency of a gene transmitted through individuals with 2n = 21 to 23 ranged from 8.7 to 10.6% in the backcross generation [51]. Contrastingly, the triploid AAC hybrids can transmit higher rates of 2n = 20 to 24 and 34 to 38, depending on the female parent. The number of chromosomes transmitted in the hybrid was found to be incomplete because a C chromosome had been introgressed onto another A or C chromosome [52]. Meanwhile, assessed from nuclear DNA content, the ploidy of the BC_1_F_1_ generation changed towards that of *B. rapa* [9]. However, it differed from *B. rapa*, indicating a small portion of the C genome, possibly as few as one or two chromosomes were present in the first meiotic division that gave BC_1_F_1_ plants. In the case of continuous backcrosses for BC_2_F_2_, the ploidy is stable after an intermating generation of BC_2_F_2_ Bulk [32].

## 4. Genetically Modified Herbicide Resistance Traits

The effects of hybridization will vary by trait, with certain qualities being more likely to promote weediness or invasiveness, and leading to reduced fitness in hybridization and introgression than others, details provided in Table 1. The *CP4 EPSPS* gene (5-enolpyruvulshikimate-3 phosphate synthase) in transgenic *B. napus* c.v. GT73 decreases binding affinity for glyphosate, conferring increased tolerance to glyphosate herbicide, and the *GOX* gene, which carries glyphosate oxidase, confers tolerance to glyphosate herbicide by degrading glyphosate into aminomethylphosphonic acid (AMPA). The traits were used in many studies [31,36,37]. The *bar* gene is responsible for phosphinothricin (PPT) tolerance in *B. napus* cv. Drakkar. This gene encodes an acetyltransferase that acetylates the free NH_2_ group of PPT to inactivate it. PPT inhibits glutamine synthetase, resulting in rapid ammonia accumulation and cell death [26,34,35]. The two gene constructs in *B. napus* cv. Westar include the *Bt cry1 Ac* gene, which is highly resistant to common defoliating lepidopterans such as the diamond black moth [53], and a plasmid containing GFP fluorescence was used to detect visual assay transgenic lines of GFP/BT events (GT1-9) [9,11,25,30,32,46] and another variant of GFP *mGFP5er* [10,38,54]. On another perspective, shortening flowering time to add early flowering genes (*BrAGL20*) with herbicide-resistance (*bar*) and hygromycin-resistance gene (*hpt*) in *B*. *napus* cv. “Youngsan” [4,55]. Subsequently, transgene mitigating genes (Δ*gai* as intact tandem genes) were inserted into *B. napus* cv. Westar herbicide resistance ahas^R^ (acetohydroxy acid synthase; conferring resistance to imidazolinone herbicides) [33,56].

## 5. Fate of Transgenes in Interspecific Hybrid Plants

The potential risk of transgenic *B. napus* plants with *B. rapa* weedy or cultivars, the gene frequency or gene transmission rate is inevitable for the subsequent generations and is very complex due to their chromosome numbers and fitness of the progenies. Metz et al. **[35]** was evaluated in the backcross population from BC_1_ to BC_4_ under selection pressure. The BC_4_ generation maintains a 10% frequency of transgene plants, which indicates that a large resource of transgenic herbicide tolerance may unintentionally gene flow into weedy populations. The PPT-tolerant of BC_1_ and their subsequent generations of BC_2_ and BC_3_ yielded PPT-susceptible plants, which might be the transgene presence on one of the chromosomes of the C genome transmitted at a low frequency after a few generations [44]. Suggestively, transgenes can more safely integrate into the C-chromosome than into the A chromosome, which could reduce the risk of introgression in nature [57]. Subsequently, Zhu et al. [10] observed three types of genetic behavior for PPT tolerant *B. napus* when crossed with *B. rapa*. The first one is to inherit transgenes. Secondly, small portions of the seeds contained transgenes located on a non-homologous C chromosome. During successive backcrossing, the C chromosome could be lost and, thirdly, through the recombination between two genomes, the C chromosome might be incorporated into an A chromosome. Later on, Lu et al. [51] agreed with the results and proposed various statistical models with and without selection pressure. The frequency of the A chromosome transgene did not vary, and the transgene’s transmission rate in both selections was 50%. However, the C chromosome frequency varied from 9 to 40% until BC_3_, when herbicidal selection stabilized the transgene at around 6% in BC_4_ and BC_5_. Tomiuk et al. [50] have not admitted the statement of integration site without more detailed genetic information about the transgenic lines of *B. napus*. The frequency of homologous and homeologous recombination events, as well as the persistence time of transgenic A or C chromosomes in backcross populations, determines the safety of an integration site. However, the herbicide-resistant gene was transferred from *B. napus* to *B. rapa*. The transgene may integrate into the *B. napus* A-set of chromosome [41]. In the case that the transgene is found on the C genome, the transgene will be deleted or greater genomic incompatibility in the next generation, resulting in no transgenic backcrosses. This could be an important investigation option for decreasing introgression [11,35]. Another study found that a transgene carried by the C chromosome is less likely to be transferred in a *B. rapa* background than a gene carried by the A chromosome, and the chance of transfer varies across the C genome [52]. Even though it is a fact that the A and C genomes share a large degree of similarity, the safe-spot idea has been questioned by several authors, and contradicting evidence suggests that transgene insertion position may not lead to greater biosafety in terms of gene flow [9,11,50,58]. Subsequently, another piece of statistical evidence with the biased and unbiased model using a mixed population of different C chromosome numbers is the relative fitness of *B. rapa* BC_1_ and F_1_ hybrids. The possible way for introgression with the transgene in the C chromosome to have a positive effect on fitness is by making the plant herbicide-resistant. An extra chromosome leads to aneuploidy. Another way is by using homeologous recombination. The transgene on the C chromosome might be integrated into the A genome. This might happen during the F_1_ hybrid’s meiosis stage. At this stage, F_1_ hybrid introgression is substantially less likely for a transgene that is already on genome A [59].

Overall, *B. napus* is an economically important crop for improvement through the addition of commercially released transgenic traits (herbicide resistance, *Bt*, and TuMV). GMO traits could have negative effects on non-target species. Crops realize their environmental harm through gene flow and their effect on interspecific hybridization to *B. rapa* subspecies or viable seeds from transgenic hybrids. Gene flow can be widespread enough to pass genes into wild relatives even when those genes are carried on unshared non-homologous chromosomes. In some GM traits, using multiple transgenes may be difficult to detect in weed populations unless the weed populations have limiting factors such as abiotic stress or herbivores. In particular, novel genes have the potential to create weed issues by providing novel traits that enable weeds to compete better, produce more seeds, and grow widely. In the review, we mainly focus on likely crops for *B. napus* such as *B. rapa* being involved in stable introgression, F_1_ hybrids, and their selfing progenies having increased the ploidy level (enhance the plant’s evolution fitness), genetic diversity of the wild relatives, and hormonal imbalance of the seeds, such as vivipary or precocious germination effect on the seeds. This could be an environmental risk for a transgene trait to persist in nature through gene flow. To reduce the gene flow, transgenes may integrate into the C genome, the transgenic plants maintained in controlled greenhouse condition, designated experimental farm, spatial distribution, and variation between the pollen recipients.

## 6. Conclusions and Future Perspective

In conclusion, we updated the progress that has been made to date in the use of interspecific hybridization of transgenic *B. napus* and *B. rapa* wild, weed, and cultivars. The hybridization in controlled environments such as greenhouses, experimental fields, and cage setups allows the GM *B. napus* to successfully hybridize with several *B. rapa* subspecies. It can generate numerous fertile and viable generations and pass the herbicide-resistant transgene to their offspring. Artificial hand pollination with GM *B. napus* produced 100% outcrossing rate in a greenhouse environment. However, spontaneous hybridization has an outcrossing rate in the field that ranges from 0.02 to 2.78% [31,60]. In comparison to greenhouse settings, the outcrossing rate is significantly lower because of many external factors. All the way through, there is no control of the transgene spread, not even using a transgene mitigation system in crops, in nature, or in greenhouse conditions. Thus, what will be the future direction of transgenic research to continue to control or mitigate the transgene spread in wild or weed populations? Previous research indicates that greenhouse containment is the best strategy for preventing natural gene flow. Another possibility is that the transgene can be transferred into the C chromosome, which could be used to eliminate it in subsequent backcross generations. The recent technological advances in genome sequencing, genotyping-by-sequencing, transcriptomic, high throughput-phenomics platforms, machine learning algorithms using methods to discriminate the transgenic plants in fields and controlled conditions, and most recent conditionally accepted methods of genome editing are being used to develop improved crop plants with different flower or leaf colors to accumulate anthocyanin that could help to eliminate transgenic volunteers or weeds and control the gene flow.

## Figures and Tables

**Table 1:** List of studies on interspecific hybridization with *B. rapa* × GM *B. napus.*

Countries	Hybridization	Variety/Cultivar	Transgenic Traits	Growing Conditions	Pollination/Mediated	References
France, USA	*B. rapa* × *B. napus* F_1_ hybrids, BC_1_	*B. napus* (Westar); *B. rapa* (weedy; Back Bay, near Irvine, California)	Bt-transgenic (*Btcry1AC*, green fluroscent protein (GFP) *mGFP5er* gene)	Glasshouse	Hand pollination and bumble bee	[28]
Taiwan	*B. rapa* × *B. napus* F_1_ hybrids	*B. rapa* var. (Nongxing, Wansheng rape and Edible rape); *B. napus* (Var. Deza oil no. 18, Gueiza No. 4, Zhong oil No. 36, Wan oil No. 25, cultivar FTHEB1001)	Synthetic GM *B. napus*	Greenhouse	Manual pollination	[29]
USA	*B. rapa* × *B. napus* F_1_, BC_1_ and F_2_ individuals	*B. napus* (Westar GT1-9); NT: *B. napus* (cv. Westar); *B. rapa* acc.2974)	*Btcry1Ac* (GFP *mGFP5er gene*) and mitigation gene (pPZP212-*ahasR*-Δ*gai-1*) confers ALS (acetolactate synthase)	Interspecific and intraspecific, competition conditions, greenhouse and shade house	Hand crossed	[30]
South Korea	*B. rapa* × *B. napus* F_1_ hybrids	*B. napus* L. (Youngsan), *B. rapa* L. ssp. *pekinensis* ‘Jangkang’	Herbicide resistance and hygromycin resistance gene, pPBrAGL20 and pHBrAGL20	GMO greenhouse	Artificial emasculation	[4]
China	*B. rapa* × *B. napus* c.v.GT73 F_1_ hybrids	*B. napus* c.v. GT73, *B. napus* c.v.Ms8x Rf3, *B. napus* c.v. Zhongyou 821 (CK, control); *B. rapa* L. ssp *pekinensis* Olsson (60), *chinensis* var. *chinesis* Kitam (33), *chinensis* var. *purpurea* Mao (4), *chinensis* var. *parachinensis* Tsen et Lee (10), *chinensis* var. *rosularis* Tsen et Lee (6), *chinensis* var. *oleifera* (3), *raifera* Matzg (2)	Glyphosate tolerant, Phosphinothricin tolerant	Greenhouse	Artificial emasculation and Spontaneous outcrossing	[31]
USA	*B. rapa* × *B. napus* F_1_ and BC_1_ F_1_, BC_2_ F_2_	*B. rapa* wild accession, *B. napus* cv. Darkkar GT 1-9	*Btcry1Ac*, GFP	Greenhouse	Houseflies	[32]
Israel	*B. rapa* × *B. napus*, F_1_ and F_1_BC_1_	T_1_*B.napus* L. cv. Westar (16 transgene mitigating (TM) lines) and J7, J9 and J16 independent lines, *B. rapa* (#2974)	pPZP212-*ahas*R-Δ*gai*-1 (TM 1), herbicide resistance	Glasshouse	Manual pollination	[33]
Canada	*B. rapa* × *B. napus* GT lines F_1_ and BC_1-4_	*B. napus* c.v. Westar (GT1-9); *B. rapa* 2974 and 2975, CA	Bt-transgenic (*Btcry1AC*, GFP *mGFP5er* gene)	Growth chamber	Manually emasculated	[10]
Denmark	*B. rapa* × *B. napus* F_1_ hybrids and BC_1-3_	*B. napus* Drakkar 93B1104, *B. rapa* BC25 (wild population, Denmark)	Glufosinate resistance, *neo* genes	Growth rooms	Bumblebees semi natural	[34]
USA	*B. rapa* × *B. napus*	seven T_3_ *B. napus* L. cv. Oscars 48,52,96,124, Westar:45,58,63; *B. rapa* weedy (CA), (MT)	*BrCry1Ac*	Growth chamber	Hand crossed	[9]
USA	*B. rapa* × *B. napus* F_1_ hybrids, BC_1_	T_1_ *B. napus* cv. Westar (GT1-9)	*BtCry1Ac*, *mGFP5er* gene	Controlled condition	Agrobacterium	[11]
Netherlands	*B. rapa* × *B. napus* F_1_, BC_1_-_4_	*B. rapa chinensis*, *B. rapa pekinensis*, *B. napus* cv. Drakkar	PPT, bar	Pollen cage at greenhouse	Emasculated	[35]
USA	*B. rapa* × *B. napus* F_1_ hybrids	*B. napus* RaideRR GT73, weedy *B. rapa* USDA-GRIN (PI 549154)	*CP4 EPSPS, GOX* and *Cry1Ac*	Glasshouse and outdoor mesocosms	Houseflies	[36]
USA	*B. rapa* × *B. napus* F_1_ and BC_1_	*B. napus* RaideRR GT73, weedy *B. rapa* USDA-GRIN (PI 633155)	*CP4 EPSPS, GOX*	Outdoor mesocosms	manual pollination	[37]
Denmark	*B. rapa* × *B. napus* F_1_, BC_1_,	*B. napus* ssp. *oleifera* (DC) var. Darkkar, NMS1, NMS1 and RF1	*bar* (barnase and barstar) encoding PAT resistant to PPT	Growth chamber to conviron growth cabinet to field	Random pollination	[26]
USA	*B. rapa* × *B. napus* F_1_, Bc_1_	T_0_ *B. napus* cv. Westar (GT2-4, GT8-9 and GFP1-3), *B. rapa* (wild relatives *Br* CA, *Br* QC-2974, *Br* QC-2975)	*BtCry1Ac, mGFP5er* gene	Field to greenhouse	spontaneous	[38]
Russia	*B. rapa* × *B. napus*, BC	NT *B. napus* cv. Ratnik, Belinda, Heros	Hygromycin phosphotransferase (HPT)	Field to greenhouse	spontaneous	[39]
France, USA	*B. rapa* × F_1_ hybrids,	*B. napus* ssp. oleifera, *B. rapa* (weed)	Bt-transgenic (*Btcry1AC*, GFP *mGFP5er* gene)	Greenhouse with microcosms	spontaneous	[25]
Canada	*B. rapa* × *B. napus* F_1_ hybrids	*B. napus* (HR 45A51, 45A50 and Westar, GT-2,6,7,8 and 9 with GP); *B. rapa* wild	*BtCry1Ac*, *mGFP5er* gene, HR,	Field experimental farm	spontaneous	[11]
Canada	*B. rapa* × *B. napus* field border plants	*B. rapa* QC-9039, QC-9047; *B. napus* (Glyphosate resistant)	Glyphosate resistant	Field experimental farm with two different sites	spontaneous	[40]
Denmark	*B. rapa* × *B. napus*, BC_1-2_	*B. napus* (Basta), *B. rapa* (weedy)	Basta herbicide tolerance	Field	spontaneous	[8]
Canada	*B. rapa* × *B. napus* F_1_ and BC_1_	*B. napus* 45A51 HR glyphosate resistant (volunteer of CP4 EPSPS), *B. rapa* (QC-9039, QC-9047)	HR glyphosate	Commercial field	spontaneous	[41]

## Data Availability

Not applicable.
